# Advances in novel biomaterials combined with traditional Chinese medicine rehabilitation technology in treatment of peripheral nerve injury

**DOI:** 10.3389/fneur.2024.1421772

**Published:** 2024-06-13

**Authors:** Xinhao Liu, Zekai Hu, Yixiao Huang, Lelun Hu, Jinnuo Lu, Mengning Chen, Han Xue, Shujie Ma, Jie Wan, Jun Hu

**Affiliations:** ^1^The Second Rehabilitation Hospital of Shanghai, Shanghai, China; ^2^Shanghai University of Traditional Chinese Medicine, Shanghai, China; ^3^Department of Acupuncture, Shanghai Shuguang Hospital Affiliated to Shanghai University of Traditional Chinese Medicine, Shanghai, China

**Keywords:** novel biomaterials, traditional Chinese medicine, rehabilitation, peripheral nerve injury, microfluidic chip

## Abstract

Peripheral nerve injuries (PNI) represent one of the primary neuropathies leading to lifelong disability. Nerve regeneration and targeted muscle atrophy stand as the two most crucial factors influencing functional rehabilitation post peripheral nerve injury. Over time, traditional Chinese medicine (TCM) rehabilitation approaches such as acupuncture, Tuina, and microneedles serve as pivot means to activate the regeneration of injured nerve Schwann cells. By promoting axon regeneration, these approaches can accomplish nerve repair, reconstruction, and functional rehabilitation. Although TCM rehabilitation approaches have clinically demonstrated effectiveness in promoting the repair and regeneration of PNI, the related molecular mechanisms remain unclear. This significantly hampers the application and promotion of TCM rehabilitation in PNI recovery. Therefore, deeply delving into the cellular and molecular mechanisms of TCM rehabilitation technologies to foster nerve regeneration stands as the most pressing issue. On the other hand, in recent years, novel biomaterials represented by hydrogels, microfluidic platforms, and new chitosan scaffolds have showed their unique roles in treating various degrees of nerve injury. These methods exhibit immense potential in conducting high-throughput cell and organoid culture *in vitro* and synthesizing diverse tissue engineering scaffolds and drug carriers. We believe that the combination of TCM rehabilitation technology and novel biomaterials can more effectively address precise treatment issues such as identification of treatment target and dosage control. Therefore, this paper not only summarizes the molecular mechanisms of TCM rehabilitation technology and novel biomaterials in treating peripheral nerve injury individually, but also explores the research direction of precise treatment by integrating the two at both macro and micro levels. Such integration may facilitate the exploration of cellular and molecular mechanisms related to neurodegeneration and regeneration, providing a scientific and theoretical foundation for the precise functional rehabilitation of PNI in the future.

## 1 Introduction

The peripheral nervous system (PNS) consists of nerve tissue located outside the brain and spinal cord. Peripheral nerve injuries (PNI) involve the structural and functional disorders of peripheral motor nerves, sensory nerves and autonomic nerves. These injuries often result in intractable neuropathic pain, with an incidence rate of approximately 13 to 23 cases per 100,000 people annually (1). Trauma, ischemia, and infection, among other factors, can lead to peripheral neuropathy, and resulting in dysfunction. Among these, penetrating injuries, traction or compression, vibration injuries and other forms of trauma are the most common causes of injury (2). The recovery of limb function following PNI serves as a criterion for assessing the efficacy of peripheral nerve repair. One significant reasons for inadequate functional recovery is the degeneration, atrophy, and fibrosis of muscles following denervation (3).

Schwann cells (SCs) are the principal glial cells of PNS. Dedifferentiation, proliferation and subsequent activation of macrophages are processes through which they are recruited to the injury site to remove myelin debris and necrotic tissue, ultimately guiding nerve regeneration (4, 5). At the same time, SCs are also responsible for the myelination of axons in PNS (6), and form nerve fibers with axons as a functional unit of PNS. Each nerve fiber is protected by the inner membrane (primarily collagen and elastic elements). The inner membrane wraps the nerve fiber groups into nerve bundles, which are enveloped by the perineuriuml (7) (primarily connective tissue). Although the axons of PNS can regenerate, achieving satisfactory results remains challenging. Excessive macrophage aggregation promotes the release of inflammatory factors, increases the level of reactive oxygen species in damaged tissues, and inhibits nerve growth (8, 9). Prolonged denervation of muscles leads to muscle paralysis and atrophy. Therefore, promoting peripheral nerve regeneration is the focus of rehabilitation after PNI, aiming to reconnect the nerve with the target organ as efficiently as possible to prevent neurological dysfunction and enhance the quality of life for patients.

Rehabilitation after PNI focuses on two aspects: promoting nerve regeneration and delaying denervated muscle atrophy. Methods to delay denervated skeletal muscle atrophy include electrical stimulation, pulsed magnetic therapy, passive exercise, ectopic foster care, gene therapy, and drugs. However, these methods have limited effects on skeletal muscle atrophy. Regarding nerve regeneration, numerous clinical and basic studies have validated the effectiveness of improved suture methods, SCs implantation, artificial nerves, nerve growth factor, gangliosides, and other drugs. Despite microsurgery’s ability to restore nerve continuity, the average regeneration rate of human peripheral nerve axons is about 1 mm/d. Long-term denervation leads to muscle atrophy, significantly limiting limb function recovery (10). Therefore, exploring the internal mechanism of functional recovery after PNI and seeking new clinical treatment strategies to delay muscle atrophy and enhance nerve regeneration are current hot topics in research.

## 2 Efficacy of Chinese medicine rehabilitation in peripheral nerve injury

**Figure 1:**
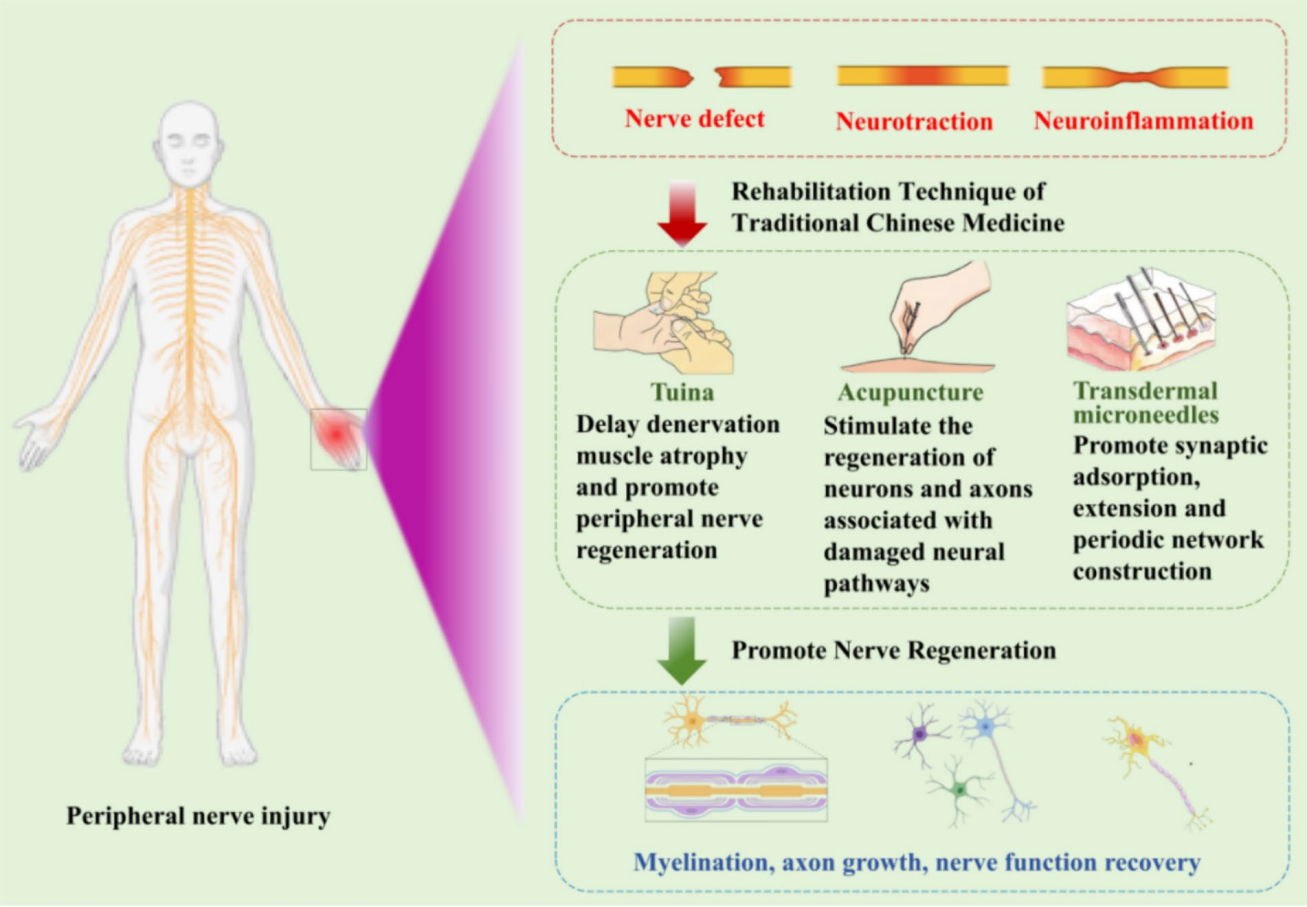
Chinese medicine rehabilitation in peripheral nerve injury.

In recent years, TCM rehabilitation therapy has received increasing attention in the treatment of peripheral nerve injuries. Its main modalities include electroacupuncture, Tuina, and Tai Chi. Among these Tuina has been widely employed as a non-invasive rehabilitation therapy technique in the treatment of peripheral nerve injuries (Figure 1).

### 2.1 Tuina

**Figure 2:**
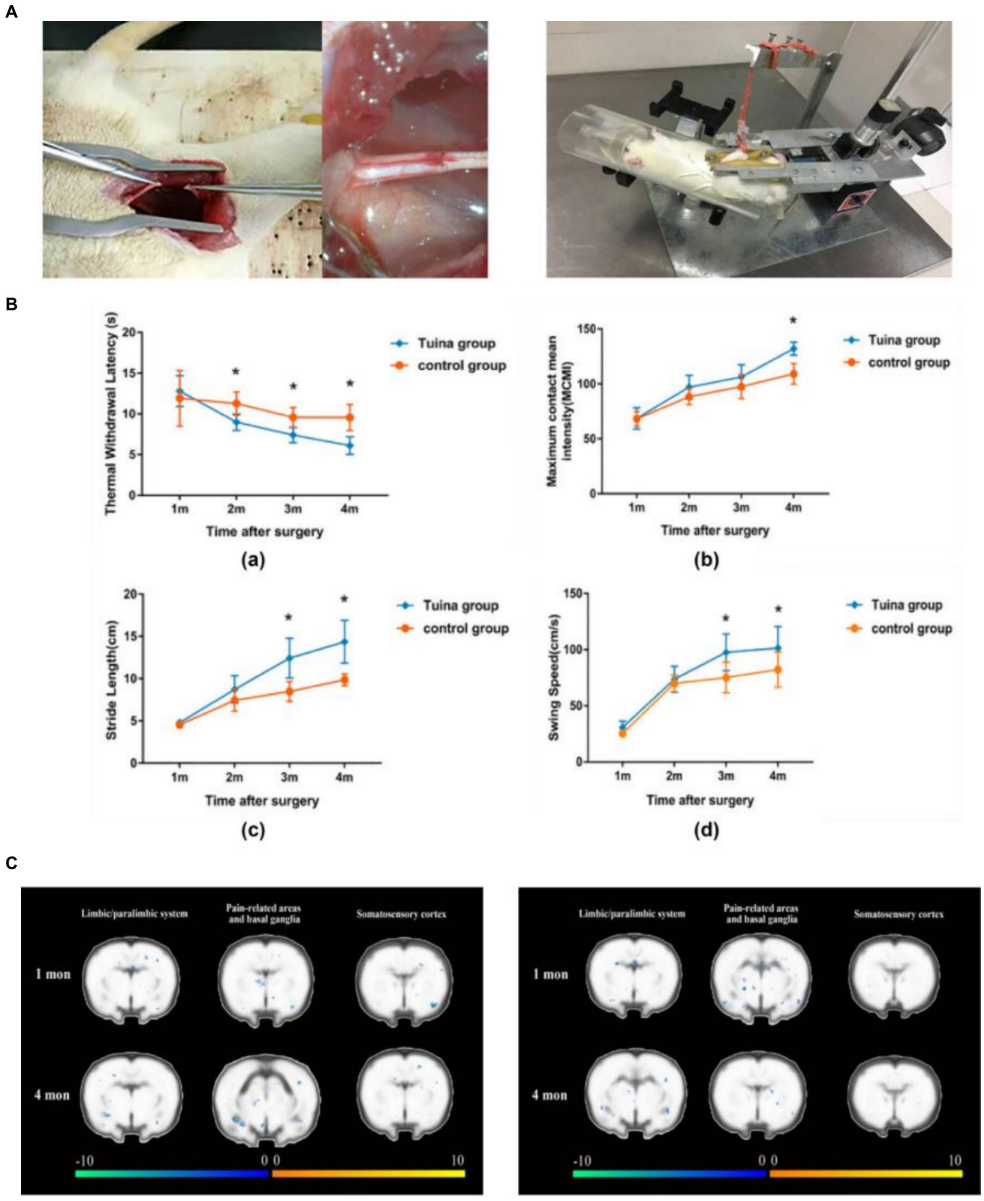
Team’s previous research - Brain mechanism study of peripheralnerve injury by Tuina. **(A)** Schematic diagram of the sciatic nerve transection wound suture model and schematic diagram of standardized Tuina therapy performed by small animal Tuina manipulation simulator (14). Copyright 2022, Chinese Association of Rehabilitation Medicine. **(B)** Comparison of behavioral tests between the Tuina group and control group at a quarter month. Thermal withdrawal latency (TWL), maximum contact mean intensity (MCMI), stride length(SL), and swing speed (SS) of rats of the Tuina group and control group at four time points (12). Copyright 2023, WILEY. **(C)** Comparison of cortical activation between the Tuina and control groups during the injured hindpaw stimulation task (12). Copyright 2023, WILEY.

Tuina is a complementary and alternative approach in rehabilitation. It consists of a number of classical maneuvers divided into four different categories: pushing and rolling, squeezing/pressing, moving joints, and vibration. It plays a special role in slowing down the atrophy of denervated skeletal muscles and promoting the regeneration of peripheral nerves (11). Our research team has recently discovered that Tuina therapy for the gastrocnemius muscle in rats with sciatic nerve injury can effectively relieve pain in the affected limb induced by peripheral nerve injury and maintain motor function. In addition, Tuina therapy reduced the activation level of pain-related brain regions and suppressed the decline in motor cortex activity induced by nerve injury, reflecting the effect of peripheral stimulation on brain plasticity (12). Based on a sciatic nerve transection model, we demonstrated that resting-state functional magnetic resonance (fMRI) performed higher ALFF values in the left somatosensory cortex of the Tuina group than in the model or sham Tuina group, suggesting that Tuina can promote adaptive changes in the somatosensory cortex and improve the recovery of local brain activity after peripheral nerve injury (13) (Figure 2). In delaying denervation muscle atrophy, by regulating muscle specific microRNA expression, massage promotes Pax 7, MyoD, MyoG pathway transcription, stimulates muscle satellite cell proliferation and differentiation, and delays denervation muscle atrophy. Additionally, by up-regulating autophagy-related factors Beclin-1, Vps34 and LC3 in mRNA level, Tuina promotes the activation of autophagy andremoves damaged organelles and protein, therefore provides certain synthetic substrate and energy for muscle fiber regeneration, thus reducing the degree of denervation skeletal muscle atrophy (15, 16).

The mechanically gated ion channel, Piezo1, converts mechanical signals received at the cell membrane into electrical or chemical signals that are transmitted to the intracellular compartment followed by causing a series of biochemical reactions. Muscle injury can be induced through apoptosis (17). Piezo1 may play a role in the apoptosis process during skeletal muscle injury, where decreased Piezo1 expression leads to more apoptotic skeletal muscle cells after injury (18). Given that Piezo1 can be activated by directly sensing changes in membrane tension, any physiological force altering membrane tension could theoretically activate Piezo1 channels (19). Piezo1 is expressed in myoblasts, myosatellite cells, and SCs (20), and its activation promotes the differentiation of myosatellite cells (21)and regulates cell apoptosis in skeletal muscle tissues. In Piezo1-deficient mice, expression levels of the pro-apoptotic factors Bax and Caspase-3 significantly upregulated, and the number of apoptotic cells increased (22). Chinese researchers have discovered that Tuina manipulation aids in the recovery of skeletal muscle motor functions and alleviates injury, a process possibly linked to the activation of Piezo1 and the inhibition of apoptosis (23). Recent studies have also shown that intermittent pressure, simulating rolling manipulation, maintains skeletal muscle calcium homeostasis and reduces skeletal muscle injury by regulating oxidative stress and lipid metabolism levels (24, 25). This is further evidence that, both inside and outside the organism, the reduction of skeletal muscle damage under pressure stimulation can help the organism to recover more rapidly after peripheral nerve injury or to perform compensatory motor functions.

In the structural aspect, Tuina manipulation maintains the ultrastructure of the key parts of the downtream pathway. This therefore promotes SCs proliferation and myelin sheaths recovery, regulates the formation of spinal cord ventral horn neurons and autophagosomes to protect neurons and axons, and enhances muscle fibers’ structural integrity. This improvement led to better motor function in the hind limbs of the sciatic nerve-injured rats, aiding in the recovery of fine motor movements and the muscle strength of hind limbs (26). It is evident that Tuina manipulation plays a crucial therapeutic role in delaying nerve regeneration, and is thereby widely used in clinical practice.

In the study of Tuina for peripheral nerve regeneration, we identified scattered research direction, mainly: (1) Screening of various Tuina techniques in clinical trials. (2) Exploring molecular mechanisms across different signaling pathways in animal experiments. (3) Selecting distinct pressure values at the cellular level. While these studies validate Tuina’s positive impact on peripheral nerve injuries, refining targeted treatments remains the focal point of ongoing research.

### 2.2 Acupuncture

As one of the most widely used therapeutic modalities for the clinical treatment of peripheral nerve injury, acupuncture has a benificial regulatory effect on peripheral nerve injury through the selection of acupoint acupuncture or special acupuncture techniques This can effectively promote nerve excitation and conduction to alleviate the local inflammation of peripheral nerves, promote the normal operation of the blood circulation, and accelerate the recovery of nerve paralysis (27). At present, the therapeutic approaches of acupuncture treatment of peripheral nerve injury aims to improve the microenvironment of nerve cell growth. By stimulating the damaged nerve pathway, it promotes reconstruction of neurons and regeneration of axon, which in turn contributes to the recovery of nerve function. Its therapeutic mechanisms involve a number of signaling pathways: (1) Notch signaling. It is the most common one in the treatment of peripheral nerve injury. Its mechanisms can primarily divide into two aspects: first, the activation of the Notch pathway signifies important changes in SCs. An interruption in the expression of Notch intra-cellular domain 1 (NICD) in this pathway leads to Schwann cell inactivated, halting the nerve myelin regeneration process. Therefore, increasing the expression of NICD in the Notch pathway after peripheral nerve injury canrapidly activate Schwann cells, which is the key to recovering from peripheral nerve injury. Second, the Notch signaling pathway can mediate several stem cell differentiation processes and plays a crucial role in maintaining stem cell pheynotype. In the nervous system, it mediates the neuronal direction differentiation of mesenchymal stem cells (MSCs), promoting nerve injury recovery (28). Using bone marrow MSCs in treating peripheral nerve injury, inhibition of the Notch signaling pathway can activate nerve regeneration and reduce secondary injury damage to the body. Chinese scholars have regulated receptor expression in the Notch signaling pathway by acupuncture at the acupoints of the governor vessel. This inhibited Presenilin1 expression, downregulated the expression levels of the pathway-related downstream genes Hes-1 and Hes-5, and facilitated the repairing effect in rats (29, 30). (2) The mitogen-activated protein kinase (MAPK) pathway is a primary pathway related to peripheral nerve injury. Acupuncture can reduce the secretion of inflammatory cytokines by inhibiting both the nuclear translocation of NF-κB p65 and the expression of p38MAPK, which improves the condition of over-activated microglia. In studies of acupuncture points for Chinese medicine rehabilitation, Zusanli, Quchi, Shenting, and Baihui were found to effectively down-regulate key targets in the TLR4/MAPKs/NF-κB signaling pathway following nerve injury. Their action mechanism is likely related to the modulation of NF-κB-mediated apoptosis closely (31). Acupuncturing at Zusanli and Quchi points can mitigate glutamate accumulation caused by peripheral nerve injury, lessen neurotoxicity from its over-enhancement, and accelerate the repair process. This is achieved by up-regulating AMP-activated protein kinase a (AMPKa) phosphorylation in the motor cortex, somatosensory cortex, and caudal shell nucleus (32). (3) Among the four pathways related to the Wnt signaling pathway, the Wnt-β-catenin one is most closely related to the proliferation and differentiation of neural stem cells. Acupuncture on the governor vessel can up-regulate the expression of Wnt1 and Wnt3a in the organism. It inhibits the phosphorylation and ubiquitination of β-catenin by regulating their expression levels, thus accelerating the proliferation and differentiation of local stem cells in favor of nerve repair and regeneration (33). Acupuncture of Dazhui and Mingmen acupoints can increase the expression level of β-catenin in the Wnt1, Wnt3a and Wnt-3-catenin signaling pathway in the organism. It protects the related limb motor function of rats with a nerve injury model (34).

Although the effectiveness of acupuncture in the rehabilitation of peripheral nerve injury has been clinically recognized, it can be used not only as an adjunctive treatment for rehabilitation after peripheral nerve injury, but also for preventive intervention for nerve repair. However, according to animal experiments results, acupuncture therapy still lacks a clear mechanism of action. Some open questions still remain, including the scattered mechanism interpretation, inconsisitent acupoints as well as acupuncture dose selection during TCM clinical treatment, thereby standardized treatment protocols are in blank. Future research directions and challenges include: (1) regulating both intra- and extracellular signaling within the peripheral and central nervous systems via stimulation of meridian acupoints; (2) assessing to which extent acupuncture can elicit the body’s self-regulatory mechanisms to influence nerve self-repair; (3) developing a standardized rehabilitation protocol for acupuncture treatment of peripheral nerve injuries utilizing novel technologies.

### 2.3 Microneedles

Microneedles are often used in TCM to treat acupuncture points on the head and face, with slight punctures at different points. They are commonly used to treat neurological disorders. With the progress of modern science, microneedles are now primarily used as a minimally invasive transdermal drug delivery device. They are consisted of neatly arranged micro-needles with a length of 20 ~ 3,000 μm, capable of delivering drugs to the subcutaneous area in a minimally invasive, safe, and painless manner (35). There are mainly four types: solid microneedles (36), coated microneedles (37), hollow microneedles (38), and dissolvable microneedles. Compared with the aforementioned types, dissolvable microneedles, are more biocompatible and offer advantages such as good biocompatibility, simple operation, minimal tissue damage, no inflammatory reaction, and controllable drug release rate, thus being the preferred choice for long-term treatment (39, 40). Although microneedle monotherapy does not provide good rehabilitation of PNI, its combination with novel biomaterials promotes nerve regeneration. Designing implantable neural devices in the form of miniature cones at the neural connection site can significantly enhance neuronal synaptic adsorption, extension, and cyclic neuronal network construction, offering a new method for neuronal network reconstruction and functional modulation (41).

Despite the obvious intervention of microneedles in the existing novel biomaterials for the treatment of peripheral nerve injury, microneedles are more often used only as a medium of drug delivery. How to combine acupoints with microneedles to exert their TCM advantages for the treatment of peripheral nerve injury needs to be clarified now.

## 3 Efficacy of novel biomaterials in peripheral nerve injury

**Figure 3:**
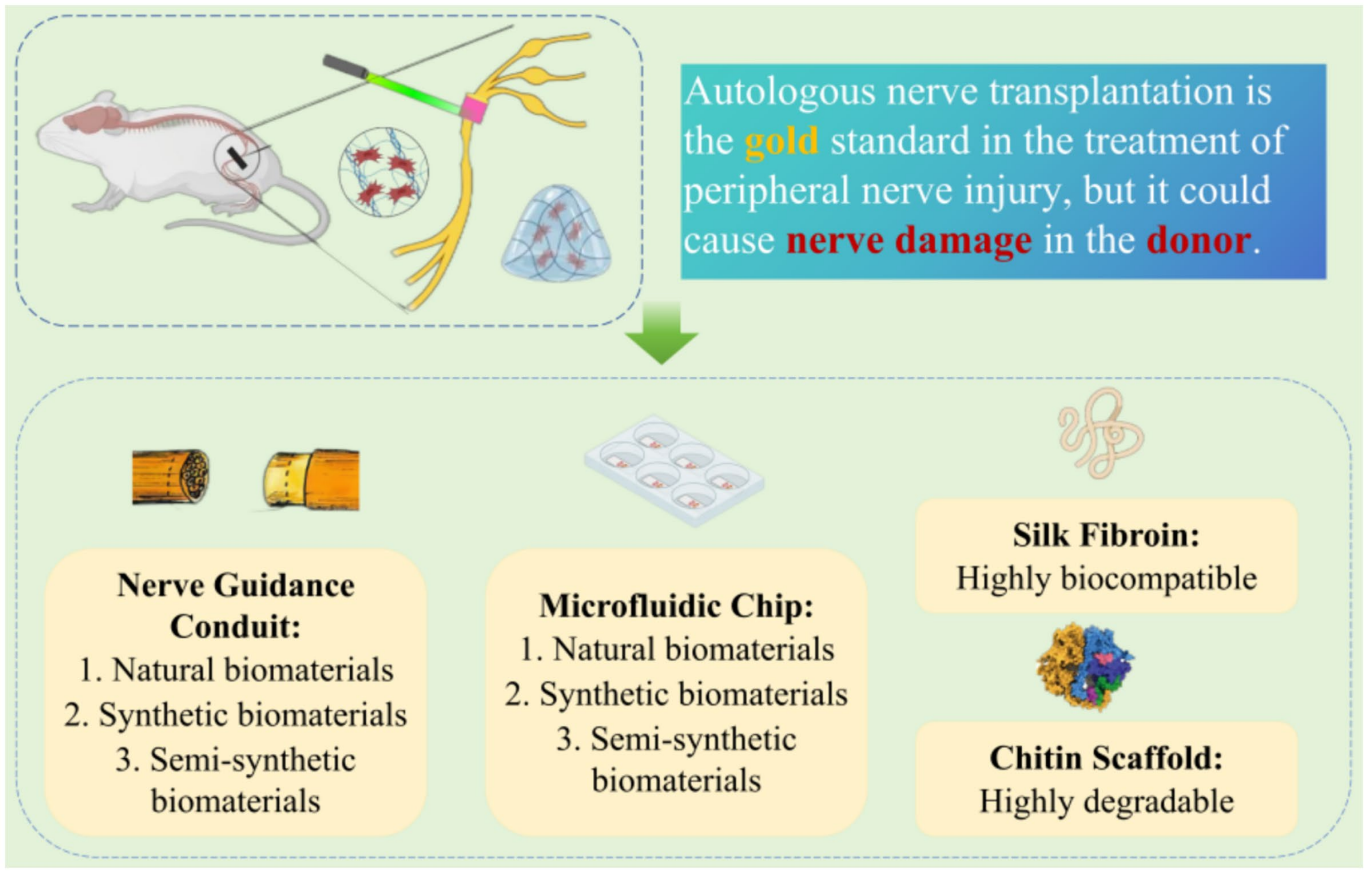
Application of novel biomaterials in peripheral nerve injury.

PNI can be classified as proximal and distal injuries based on the severity of the rupture. In clinical treatment, it is common to use microscopic surgery to suture the severed ends of nerves. However, for larger peripheral nerve defects (> 1 cm in rats and > 3 cm in humans), autogenous nerve transplantation (ANT) is considered the gold standard treatment. Contralateral seventh cervical nerve (CC7) cross-transplantation surgery provides effective, safe, and stable functional improvement for patients with unilateral arm spastic paralysis for over 5 years. Compared to non-transplanted patients, those who undergo CC7 cross-transplantation show significantly reduced degrees of arm spasticity and notable functional improvement during both 1- and 2-year follow-up periods (42, 43). However, ANT has its limitations and may lead to functional nerve damage in the donor. Therefore, novel biomaterials derived from synthetic or natural sources have emerged as a new rehabilitation therapy method for promoting nerve regeneration (44) (Figure 3).

Depending on the severity of the injury and considering the characteristics of various materials, different types of novel biomaterials can be applied. For smaller nerve defects, novel nanomaterials can promote the recovery of PNI. Cerium oxide nanoparticles promote the recovery of rat sciatic nerve injuries caused by compression. High doses of cerium oxide nanoparticles significantly enhance the regeneration rate of the injured sciatic nerve, increasing the number of myelinated fibers and the thickness of the myelin sheath in compression-induced sciatic nerve injuries. Therefore, these nanoparticles can serve as a new therapeutic reagent for nervous system regeneration (45).

When the nerve injury is on a broader scale, tissue-engineered nerve grafts (TENG) made from novel biomaterials can be utilized to promote regeneration of the damaged nerves. According to cholera toxin B subunit (CTB) tracing, sciatic nerve functional index, electromyography, and immunofluorescent staining of regenerated nerves and motor endplates, the regeneration of the sciatic nerve was observed. It was found that the combination therapy of a novel chitosan scaffold (CS) loaded with basic fibroblast growth factor (bFGF) significantly improved the outcome in rats with 2 cm sciatic nerve defects. Evaluation of its mechanical properties using a tensile testing machine and nanoindenter showed that, after 12 weeks of surgery, the structure and mechanical properties of bFGF-CS were conducive to sciatic nerve regeneration. This demonstrates that bFGF-CS can promote rat sciatic nerve regeneration (46). Chitosan nanofibers loaded with biologically active peptides RGI and KLT, simulating brain-derived neurotrophic factor (BDNF) and vascular endothelial growth factor (VEGF), respectively, were prepared using a combination of electrospinning and mechanical stretching methods. In this manner, chitosan RGI/KLT nerve grafts for repairing 15 mm sciatic nerve defects in rats was constructed. Twelve weeks after transplantation, chitosan RGI/KLT nerve grafts significantly promoted nerve regeneration and functional recovery in rats (47).

### 3.1 Nerve guidance conduit

Nerve guidance conduits (NGC) are primarily used in the repair of larger nerve defects. Currently, commonly used materials for nerve conduits include natural biomaterials, synthetic biomaterials, or semi-synthetic biomaterials. NGCs are primarily tubular structures that can be filled with other biomaterials according to the needs of the condition. They provide a physical channel for the regeneration of peripheral nerves by supporting the structure and connecting the damaged area. NGCs made from natural or synthetic biomaterials have become alternatives to autogenous nerve grafts, successfully bridging peripheral nerve defects and promoting nerve repair and regeneration (48).

Nerve guidance conduits play a crucial role in the treatment of diseases and the process of nerve repair. Their biocompatibility and degradability are key factors. Ideally, NGCs allow nutrients to enter the conduit to support cell growth while promptly eliminating metabolic waste (49). Materials of natural origin, such as type I collagen, laminin, fibronectin, and chitosan, have been used to prepare nerve scaffolds and replace autogenous nerve grafts due to their excellent biocompatibility (50).

To improve the effectiveness and safety of NGCs in peripheral nerve injury rehabilitation, advancements have been made in three main areas: (1) Biomimetic design: Biomimetic approaches have been successfully used to develop tubular constructs, focusing on the utilization of extracellular matrix and biomaterials to fully exploit their functions and create NGCs with mechanical characteristics resembling peripheral nerves (51). These biomaterials are derived from natural sources, such as collagen from bovine tendons or calf skin, chitosan, etc. (52). (2) Scaffold modification: Peripheral nerves are mainly composed of highly ordered bundles of axons, forming tubular or rod-shaped 3D structures. Repairing peripheral nerves requires 3D structures to guide cell growth directionally. Proper modification of NGC scaffolds can enhance their repair effects (53, 54). (3) Surface modification: Laminin and fibronectin are two basal membrane proteins crucial in the pathological development process following peripheral nerve injury, as they can bind to SCs. Therefore, surface modifications play a vital role in enhancing NGC performance post-injury (55, 56).

### 3.2 Silk fibroin

Silk fibroin (SF) is a tissue engineering material with high biocompatibility. After using electrospun SF fibers to bridge a 30 mm gap in the dog sciatic nerve and 12 months post-operation, the weight-bearing, maximum tension, and standing time of the SF electrospun fiber transplant group significantly increased compared to the non-transplanted control group. The SF electrospun fiber transplant group showed comparable compound muscle action potentials, nerve conduction velocity, and the number of sensory and motor neurons comparable to those of the autogenous nerve transplant group, proving that SF electrospun fibers are potential substitutes for autogenous nerve transplantation (57).

Bone marrow mesenchymal stem cells (BMSCs) from dogs were cultured on the surface of chitosan/SF scaffolds, then decellularized to form tissue-engineered nerve grafts (TENG) with an acellular matrix (ACM) coating, used to bridge a 60 mm gap in the dog sciatic nerve. The experimental findings showed that TENG containing ACM significantly enhanced axonal regeneration and Schwann cell proliferation. After 12 months post-transplantation, the therapeutic effects of this nerve scaffold, as determined through behavioral, functional, and histological evaluations, were found to be consistent with autogenous nerve transplantation effects (58). Moreover, TENG containing ACM showed good results in repairing the brachial plexus nerve in rats. Skin-derived precursor SCs(SKP-SCs) were cultured with SF, then decellularized to form ACM-containing SF. Ten bundles of SF were placed into conduits to prepare TENG, used to bridge an 8 mm gap in the rat brachial plexus nerve. Histological analysis performed 2 weeks post-operation showed that TENG treatment promoted axonal growth. Behavioral tests conducted 4 weeks post-operation showed that rats treated with TENG performed similarly to those treated with autologous transplantation. These results provide a theoretical basis for the clinical application of TENG containing SF (59).

### 3.3 Chitin scaffold

A new nerve graft composed of biodegradable chitin scaffolds and small autogenous nerves (SAN) can promote the regeneration of the rat sciatic nerve and myelin sheath, reduce target muscle atrophy, and facilitate nerve function recovery (60). Chitin bioconduits loaded with SAN and platelet-rich plasma (PRP) were prepared as nerve grafts to repair a 10 mm defect in the rat sciatic nerve. Twelve weeks post-operation, the sciatic nerve functional index, compound action potential amplitude, myelinated nerve fiber density, and myelin thickness all significantly increased. The density of sensory and motor neurons in the motor endplates of the group treated with chitin bioconduits loaded with SAN and PRP was higher than in the group treated with only one of them (PRP or SAN), indicating that chitin conduits simultaneously loaded with SAN and PRP had better repair effects (61).

The repair effect of acellular cauda equina allografts (ACEA) composed of chitin conduits and decellularized horse tails is effective for PNI. Compared to the sciatic nerve, the horse tail can be more effectively decellularized and contains higher amount of nerve basal lamina. ACEA can more effectively guide the axonal regeneration and migration of SCs in the cultured dorsal root ganglia than acellular sciatic nerve allografts (ASNA). *In vivo*, connecting a 15 mm defect in the rat sciatic nerve with ACEA, the regenerated nerve fibers in the ACEA group showed no significant difference compared to the autogenous transplant group 3 weeks after transplantation. Twelve weeks post-transplantation, gait analysis, neurophysiological, and histological analysis demonstrated that the repair effect of the sciatic nerve in the ACEA group was not significantly different from the autogenous transplant group, but was superior to the chitin conduit group and ASNA group. This suggests that ACEA may become a new biological material to replace autogenous transplants for the treatment of long-distance peripheral nerve defects (62).

### 3.4 Microfluidic chip

In the rehabilitation process of peripheral nerve injury, a variety of therapeutic means may be used to promote nerve repair.Thus, the establishment of an *in vitro* model is of great significance for the efficient and quantitative evaluation of the curative effect of peripheral nerve injury rehabilitation treatment. Compared with *in vivo* animal models, *in vitro* biological experiments based on microfluidic platforms offer advantages such as high throughput, humanization, and high biomimetic biology. Therefore, they are widely used in the establishment of various types of nerve injury models (63–66). The *in vitro* model constructed using microfluidic technology enables the targeting of manipulated neurons or axons while allowing real-time monitoring of their behavioral changes. Separating axons from the cell body using microfluidic techniques enables studies of neural regeneration mechanisms with specific therapeutic modalities (67, 68). Therefore, the use of microfluidic technology can construct various nerve injury models, potentially serving as a method for studying mechanisms related to nerve regeneration.

Unlike other cells in the human body, neurons form highly specific tissue structures with unique morphological and electrophysiological characteristics, making the nervous system one of the most complex systems in the human body. During nerve development, some nerve bundles are connected together through the epineurium to form nerve trunks. Complex microcirculation extends longitudinally along the nerves in the peripheral nervous system (PNS), providing oxygen and nutrient. Additionally, the endothelial cells in the microvasculature of the nerve endoneurium form the blood-nerve barrier (BNB), maintaining the inner stability of the nerves. Therefore, simulating the neural microenvironment is important for an in-depth study of the complex mechanisms of nerve injury. The use of *in vitro* models with microfluidic technology can simplify the environment of nerve cells while ensuring appropriate microenvironmental conditions. Microfluidic technology emphasizes key factors in simulating the microenvironment of stem cells and SCs such as static and dynamic kinetics, multicellular interactions, and spatial–temporal heterogeneity distribution of various signaling molecules, thereby reducing irrelevant influences and producing clearer and more reliable data (69). Currently, scholars have used ultra-slow microfluidic devices to construct gradient flows containing nutrients, growth factors, and neurotrophic factors, where stem cells differentiate into neuronal cells and SCs, and differentiated SCs can form myelin sheaths around neurons. Stem cells differentiate into neuronal cells and SCs *in vitro*, and the results show that differentiated SCs can form myelin sheaths around neurons *in vitro* (70).

The *in vivo* physiological microenvironment of neural cells, as well as the morphology and function of neural tissue, are two key elements in the establishment of microscopic models of highly biomimetic neural tissue. *In vivo*, individual neurons are embedded in larger and more complex longitudinal tissue structures, thus forming a bundle of axons or nerves. Kitagawa et al. (69) prepared alginate gel microfibers using a multilayer microfluidic device with a micronozzle array structure, which wrapped PC12 nerve cells in a parallel region composed of a soft hydrogel matrix to form groove fibers. After 2 weeks of culture, a millimeter-long intercellular network was formed, mimicking the complex nerve tract structure in the body. The fiber can be used to simulate other linear structures in the human body, and is suitable for the preparation of neural network models (69). However, with the progress of modern tissue engineering, we find that the neural network model with three-dimensional structure is closer to the human physiological environment than the two-dimensional neural network. Therefore, cerebral organoids are gradually emerging, and cerebral organoids based on microfluidic platforms can also be applied to microenvironment simulation, disease modeling, and drug screening. Wang et al. (70) used an organoid chip system to generate three-dimensional brain organoids derived from human neural stem cells hipsc. Their system provided a similar microenvironment to the brain by integrating 3D Matrixgel, fluid flow, and organized multicellular structures, demonstrating neuronal differentiation, brain regionalization, and cortical spatial organization, successfully simulating early human brain development. Noorani et al. (71) used microfluidic-based organoid chip technology to establish a new blood–brain barrier model *in vitro*, which simulated the hemodynamics and structural characteristics of cerebral microvessels that cannot be realized by traditional two-dimensional platforms. The study showed that the multicellular co-culture system has great potential in the study of central nervous system diseases.

## 4 The association between TCM rehabilitation and novel biomaterials

As one of the oldest traditional medicines of the Chinese nation, TCM has played an important role in the long history of human development. However, with the the progress of science and technology, and the continuous evolution of diseases, the limitations of traditional TCM theories gradually appear, and the unclear target mechanism and unclear effective substances limit the wider application of traditional TCM in the diagnosis and treatment of current diseases. The emergence of novel biomaterials has provided a large number of new technologies and methods for the diagnosis and treatment of various diseases, greatly expanded the means and efficiency of the diagnosis and intervention of related diseases, and thus significantly promoted their wide clinical application (72–74). The joint application of TCM and novel biomaterials provides, on the one hand, the synergistic effect of the two also significantly improves the efficiency of disease diagnosis and treatment with TCM (75, 76). In the macro aspect, graphene is combined with meridians and acupoints, and applied in the research and development of TCM meridian detector, acupoint moxibustion, Qigong, promoting the substantive research progress of TCM meridians and expanding the application scope and safety of TCM treatment methods in clinical practice (77). Chinese scholars have combined acupuncture with new biological materials to develop a real-time monitoring system of dopamine molecules and neuropeptide Y through a miniature sensor. The new needle field- effect transistor-based acupuncture microsensor is very sensitive to neurotransmitter release in the brain and has been applied in Parkinson’s disease research within a central nervous system model (78, 79).

In addition, some classic TCM rehabilitation methods, such as Tuina, may have potential synergistic effects with novel biomaterials that regulate the mechanical microenvironment of peripheral nerves and promote the repair of peripheral nerve injury. For example, an alginate-based hydrogel can achieve efficient differentiation of human neural stem cells into astrocytes. Its regulation mechanism to promote differentiation may involve the hydrogel spontaneously de-crosslinking changing its mechanical properties, transferring mechanical stimulation, activating the Piezo 1 calcium channel, promoting YAP nuclear transcription through the actin cytoskeleton, and eventually stimulating astrocyte differentiation. This provides new insights into the mechanism of mechanical regulation of astrocyte development (80). Tuina treatment involves regular mechanical stimulation, and activating the Piezo 1 calcium channel may be a key mechanism of its effect. Therefore, the mechanism of Tuina in promoting nerve regeneration may have a similar effect to that of the hydrogel’s de-crosslinking during mechanical stimulation. This similarity could play a significant role in various clinical applications of Tuina when combined with new materials.

Based on factors such as cell microenvironment and material compatibility (81), novel biomaterials have been used in the research of many basic diseases (82–85). The combination of TCM and new materials in clinical lesion positioning and other disease diagnosis/treatment scenarios is also being constantly explored. With high spatial and temporal resolution, lesion localization therapy can precisely deliver drugs to the injury site using methods such as microneedle patches. Related techniques have been applied to the localization treatment of lesions in various disease microenvironments, including tumors and various diseased organs. However, TCM theory describes microneedles, also known as “small needles,” are relatively small, their small-scale features limiting stimulation mainly to shallow tissue with weak penetration. However, in clinical practice, the deep physical barrier formed by physiological structures such as cartilage and intervertebral disc greatly hinders the implementation of lesion localization treatment. Therefore, to address this issue, scholars combine TCM classic positioning therapy acupuncture with new materials, using a composite material method for minimally invasive precision positioning of acupuncture needles and efficient drug load integration into hydrogels. This approach increases the depth of treatment and achieves precise positioning of drugs and therapy (86). Therefore, the new drug precision delivery system that combines, TCM with new materials has become a hot topic in the industry. Existing research on acupuncture with hydrogel (87, 88), as well as nanoparticles and hydrogel, has shown that these can overcome the limitations of a single drug delivery system by improving bioavailability and drug release and extending the treatment window (89, 90). Additionally, using nanocomposite hydrogel for acupoints burial with Chinese herbal medicine may offer a new method for targeted and continuous drug delivery with less side effects. Acupoint burial has many advantages in the process of continuous acupoint stimulation, reducing pain caused by frequent acupuncture, dose control, and minimal side effects in the treatment process. Therefore, increasing scholars use the method of burying new material gel into acupoints for treatment and have received satisfactory results.

## 5 Outlook

In research combing TCM with new biological material for treating peripheral nerve injuries, the application of acupuncture and new materials already has a solid foundation. However, studies on acupuncture and new materials primarily focus on physical simulation, efficacy, and depty. However, Chinese medicine is extensive and profound, with acupuncture techniques being an important part of the treatment. Acupuncture techniques include the lifting and inserting, twisting, scraping, and following methods. Properly applied acupuncture techniques can improve the “gas” feeling and achieve better therapeutic effects. In different diseases, usnew technologies and methods to study the combination of different acupuncture techniques and different properties of new materials have emegered as powerful tools, and selection of the optimal combination of acupuncture, techniques, acupuncture points and new materials will greatly improve the clinical application effect. Tuina, as the oldest treatment method of TCM, is an important supplementary and alternative therapy of contemporary medicine, and its research and application with new materials is still in the initial stage. Tuina is the use of various different techniques on the specific parts of the body or acupoints to achieve the purpose of prevention and treatment of diseases. Tuina positively impacts the entire course of peripheral nerve injury. The therapeutic effect of Tuina closely related to its mechanical parameters including type of stress, strength, angle, frequency, intervention duration, timing. However, there is still a lack of unified standards and systems to evaluate the parameters and effects of Tuina manipulation, which seriously affects the promotion and application of Tuina manipulation in peripheral nerve injury diseases. We believe that integrating tuina with new materials such as microfluidic chips, by simulating the microenvironment of peripheral nerve injury and regeneration *in vitro*, and applying tuina techniques for intervention, we can objectively and quantitatively study the effects of different tuina techniques under varying degrees of force, angle, frequency, intervention time, and treatment duration on peripheral nerve regeneration. This will enable us to identify the optimal tuina treatment protocol for the regeneration process of peripheral nerves, significantly advancing the application of tuina in the context of peripheral nerve injury and regeneration.

## Author contributions

XL: Conceptualization, Writing – original draft. ZH: Investigation, Writing – original draft. YH: Investigation, Software, Writing – review & editing. LH: Data curation, Formal analysis, Writing – review & editing. JL: Data curation, Methodology, Writing – original draft. MC: Software, Visualization, Writing – original draft. HX: Visualization, Writing – original draft. SM: Funding acquisition, Resources, Supervision, Writing – review & editing. JW: Resources, Supervision, Validation, Writing – review & editing. JH: Funding acquisition, Project administration, Resources, Supervision, Writing – review & editing.
